# Behavioral and Health Outcomes of mRNA COVID-19 Vaccination: A Case-Control Study in Japanese Small and Medium-Sized Enterprises

**DOI:** 10.7759/cureus.75652

**Published:** 2024-12-13

**Authors:** Eiji Nakatani, Hisayo Morioka, Takayuki Kikuchi, Masanori Fukushima

**Affiliations:** 1 Research Support Center, Shizuoka General Hospital, Shizuoka, JPN; 2 Health Data Science, Learning Health Society Institute, Nagoya, JPN

**Keywords:** case-control studies, covid-19, preventive health behavior, susceptibility, vaccination

## Abstract

Background

Despite ongoing waves of Coronavirus disease 2019 (COVID-19) infections, including significant surges such as the 10th wave, understanding the impact of messenger RNA (mRNA) COVID-19 vaccination on infection risk and associated behavioral changes remains crucial. This study aims to urgently evaluate the effects of mRNA COVID-19 vaccination on COVID-19 infection rates and related behaviors among participants of the Yamato Project, which includes employees of Japanese small and medium-sized enterprises (SMEs).

Methods

A case-control study was conducted using data collected from a survey administered by the Japan Small and Medium Enterprise Management Council in December 2023. Participants included individuals who were part of the Yamato Project, not necessarily limited to SME employees. The survey gathered information on demographic characteristics, COVID-19 infection status, vaccination history, health status before January 2020, and various preventive behaviors. The primary outcome was the presence or absence of COVID-19 infection. Data were analyzed using univariate and multivariate logistic regression models to calculate odds ratios (ORs) and 95% confidence intervals (CIs) for the association between vaccination status and COVID-19 infection.

Results

A total of 913 participants were included in the final analysis. The adjusted ORs for COVID-19 infection among vaccinated individuals compared to unvaccinated individuals were 1.85 (95% CI: 1.33-2.57, p < 0.001). The odds of contracting COVID-19 increased with the number of vaccine doses: one to two doses (OR: 1.63, 95% CI: 1.08-2.46, p = 0.020), three to four doses (OR: 2.04, 95% CI: 1.35-3.08, p = 0.001), and five to seven doses (OR: 2.21, 95% CI: 1.07-4.56, p = 0.033). Behavioral analysis indicated that a reduced frequency of bathing and exercising was significantly associated with higher COVID-19 infection rates (p < 0.05).

Conclusions

The study observed a higher reported incidence of COVID-19 infection among vaccinated individuals during the pandemic period, which increased with the number of vaccine doses received. This paradoxical finding may be influenced by various factors, including immune response mechanisms, such as antibody-dependent enhancement (ADE) or original antigenic sin, behavioral changes, and exposure risk. Understanding these factors is crucial for urgently enhancing public health strategies and vaccination programs.

## Introduction

The Coronavirus disease 2019 (COVID-19) pandemic, which emerged in late 2019, has profoundly impacted global health and economies. Despite widespread vaccination efforts, the pandemic continues with periodic resurgences, including significant waves like the 10th surge, which have seen increased infection rates. Vaccination campaigns have played a pivotal role in controlling the spread of severe acute respiratory syndrome coronavirus 2 (SARS-CoV-2) and reducing the severity of COVID-19 [1,2]. Messenger RNA (mRNA) vaccines developed by Pfizer-BioNTech and Moderna have demonstrated high efficacy in preventing symptomatic infection, hospitalization, and death [3,4].

Despite the overall success of vaccination programs, breakthrough infections were reported in fully vaccinated individuals, especially with the emergence of new variants of concern [5,6]. These events raised questions about factors influencing infection risk post-vaccination, including waning immunity and behavioral changes [7,8]. Moreover, the impact of vaccination on individual preventive behaviors has been a subject of research, with some studies suggesting that vaccinated individuals might perceive themselves as less at risk and consequently reduce adherence to non-pharmaceutical interventions (NPIs) [9].

Understanding the interplay between vaccination status, preventive health behaviors, and infection risk remains important, especially in the face of ongoing infection waves. Insights gained can inform immediate public health strategies and preparedness for potential outbreaks. Small and medium-sized enterprises (SMEs) constitute a significant portion of the workforce in Japan, accounting for about 70% of employment. Employees in SMEs may face unique challenges, such as close working environments and limited resources for implementing NPIs, which could influence infection risk and vaccination behavior during the pandemic.

This study aims to evaluate the effects of mRNA COVID-19 vaccination on COVID-19 infection rates and related preventive behaviors among Yamato Project participants and employees of Japanese SMEs during the pandemic period. By investigating the association between vaccination status, preventive health behaviors, and infection risk, we seek to provide insights that can enhance public health policies and workplace safety measures.

We also seek to examine behavioral changes attributable to vaccination among employees of Japanese SMEs, such as participants of the Yamato Project. Further, we endeavor to clarify whether our findings may be generalized to similar workforce populations outside of Japan.

## Materials and methods

Study design and population

A case-control study was conducted to investigate the impact of mRNA COVID-19 vaccination on the risk of contracting COVID-19 among Yamato Project participants and employees of Japanese SMEs. Data were collected through a survey administered by the Japan Small and Medium Enterprise Management Council (JSMEC) in December 2023.

Random sampling methods were not used. The dataset might not have fully represented all SME employees in Japan. Still, it included multiple types of enterprises from different geographic locations and industries, aimed at achieving a diverse sample. Thus, the study team believed that the participants reasonably approximated the broader workforce of SMEs in Japan. The inclusion criteria were participation in the Yamato Project and/or being an SME employee and completing the survey with valid responses regarding COVID-19 infection status and vaccination details. Participants with missing data on these key variables were excluded from the final analysis.

For the case-control study design, cases were defined as participants who self-reported having contracted COVID-19, while controls were those who reported no history of infection. No matching procedures were conducted; rather, all eligible participants meeting the respective criteria for cases or controls were included in the analysis.

Data collection

The questionnaire did not undergo pre-testing to assess clarity and relevance. However, content validity was ensured through expert review by epidemiologists and public health professionals. The questionnaire collected information on demographic characteristics (age and gender), COVID-19 infection status, vaccination status (including the number of vaccine doses received), health status before January 2020 (presence of any chronic health conditions), and various preventive behaviors. Preventive behaviors assessed included regular gargling, mask-wearing, bathing frequency, avoiding crowded places, room ventilation, eating habits, sleep patterns, exercise habits, and maintaining humidity in living spaces.

Data were collected using paper-based questionnaires, and a 93% response rate was achieved. The collected questionnaires were double-entered by EPS Corporation, Tokyo, Japan, to ensure data accuracy, and the data were compiled into a database for analysis.

Outcome measures

The primary outcome was the presence or absence of self-reported COVID-19 infection. The secondary outcome was the frequency of COVID-19 infections, defined as whether the participant had contracted the virus more than once.

Statistical analysis

Continuous variables were reported as means and standard deviations, while categorical variables were reported as frequencies and percentages. Independent t-tests were employed to compare continuous variables between groups, and Chi-square tests were used to compare categorical variables.

Univariate and multivariate logistic regression analyses were conducted to calculate odds ratios (ORs) and 95% confidence intervals (CIs) for the association between vaccination status (and the number of vaccine doses) and COVID-19 infection. The multivariate logistic regression models were constructed to adjust for potential confounders identified through univariate analyses and clinical relevance. Variables included in the models were age, gender, pre-existing health conditions before January 2020, and preventive behaviors such as bathing and exercising. Confounders were controlled by including them as covariates in the models, allowing us to estimate adjusted ORs and 95% CIs for the association between vaccination status and COVID-19 infection. The Wald test was used to determine the statistical significance of the associations.

All analyses were performed using SAS statistical software version 9.4 (SAS Institute Inc., Cary, NC, USA). A p-value of less than 0.05 was considered statistically significant.

## Results

Background distribution of participants

Out of 931 survey responses, 913 participants were included in the analysis after excluding 18 with missing data on COVID-19 infection status and vaccination details. Among these, 433 (47.4%) reported having contracted COVID-19, while 480 (52.6%) had not.

Demographic characteristics

The demographic characteristics of the participants are summarized in Table 1. The mean age of participants who contracted COVID-19 was significantly lower (46.75 ± 15.11 years) compared to those who did not (51.57 ± 15.67 years) (p < 0.001). The proportion of males was higher in the infected group (55.9%) than in the non-infected group (48.1%) (p = 0.023).

**Table 1:** Patient’s characteristics COVID-19, Coronavirus disease 2019

Variable	Category	COVID-19 infection		p-value
		Yes	No	
		n = 433	n = 480	
Age (years)		46.75 (15.11)	51.57 (15.67)	<0.001
Sex (%)	Men	242 (55.9)	230 (48.1)	0.023
Vaccination status (%)	Yes	222 (51.3)	187 (39.0)	<0.001
Number of vaccine doses (%)	0	211 (48.8)	293 (61.4)	0.001
	1	7 (1.6)	7 (1.5)	
	2	77 (17.8)	57 (11.9)	
	3	83 (19.2)	57 (11.9)	
	4	24 (5.6)	26 (5.5)	
	5	15 (3.5)	11 (2.3)	
	6	5 (1.2)	14 (2.9)	
	7	10 (2.3)	12 (2.5)	
Health status before January 2020 (%)	Consulted a doctor (outpatient/inpatient)	34 (8.2)	34 (7.9)	0.77
	Had health issues but did not consult a doctor	14 (3.4)	11 (2.5)	
	Healthy	369 (88.5)	387 (89.6)	
Preventive actions in daily life				
Gargling (%)	Yes	188 (43.4)	185 (38.5)	0.153
Mask-wearing (%)	Yes	91 (21.0)	108 (22.5)	0.644
Bathing (%)	Yes	166 (38.3)	223 (46.5)	0.016
Avoiding crowds (%)	Yes	64 (14.8)	77 (16.0)	0.664
Ventilation (at the workplace, etc.) (%)	Yes	121 (27.9)	113 (23.5)	0.148
Eating habits (%)	Yes	240 (55.4)	314 (65.4)	0.003
Sleep habits (%)	Yes	253 (58.4)	300 (62.5)	0.234
Exercise (%)	Yes	170 (39.3)	230 (47.9)	0.01
Humidity control (%)	Yes	62 (14.3)	73 (15.2)	0.776

Vaccination status and COVID-19 infection

A higher percentage of participants who contracted COVID-19 had received at least one dose of the vaccine (51.3%) compared to those who did not contract the virus (39.0%) (p < 0.001). The odds of contracting COVID-19 were higher among vaccinated individuals compared to unvaccinated individuals, with an unadjusted OR of 1.65 (95% CI: 1.27-2.14, p < 0.001) and an adjusted OR of 1.85 (95% CI: 1.33-2.57, p < 0.001).

Impact of number of vaccine doses

The impact of the number of vaccine doses on COVID-19 infection risk is shown below. Participants who received one to two doses had an adjusted OR of 1.63 (95% CI: 1.08-2.46, p = 0.020); those who received three to four doses had an adjusted OR of 2.04 (95% CI: 1.35-3.08, p = 0.001); and those who received five to seven doses had an adjusted OR of 2.21 (95% CI: 1.07-4.56, p = 0.033), indicating a higher reported risk of infection with an increasing number of doses.

Secondary outcomes

Among participants who had contracted COVID-19 more than once (n = 86), there was no significant difference in vaccination rates compared to those who were infected only once (vaccination rate: 39.5% vs. 46.6%, p = 0.260). The adjusted OR for reinfection was 1.44 (95% CI: 0.81-2.55, p = 0.221).

Behavioral factors

Various behavioral factors were analyzed to understand their association with COVID-19 infection. Significant differences were observed in practices such as bathing frequency (p = 0.016) and exercising (p = 0.010), with lower rates of infection observed in participants who practiced these behaviors more frequently.

## Discussion

This study provides insights into the association between mRNA COVID-19 vaccination and self-reported COVID-19 infection among employees of Japanese SMEs during the pandemic period. Interestingly, vaccinated individuals reported higher incidences of COVID-19 infection compared to unvaccinated individuals, with the reported risk increasing alongside the number of vaccine doses received. Additionally, significant differences in preventive behaviors - such as bathing frequency and exercising - were noted between those who contracted COVID-19 and those who did not.

Our study employed robust statistical approaches, including multivariate logistic regression models, to identify associations between vaccination status and infection risk while accounting for potential confounders. The high response rate (93%) suggested that the findings were based on a substantial portion of the target population, thereby increasing the reliability of our results. Furthermore, by examining behavioral factors and vaccination data, we provided a more nuanced perspective on the interplay between immunization, preventive measures, and subsequent infection risk.

Possible explanations (behavioral factors and risk compensation)

One potential explanation for these findings is behavioral risk compensation. Vaccinated individuals may have perceived themselves as less susceptible to infection and consequently reduced adherence to NPIs, such as mask-wearing, social distancing, and hand hygiene during the pandemic [9]. This could increase their exposure to the virus. Wright et al. [10] found that risk compensation behaviors can mitigate the benefits of vaccination programs if not adequately addressed through public health messaging.

While we suggested that vaccinated individuals may have reduced their adherence to preventive measures, such as mask use and distancing, we did not rigorously quantify this behavior within our data. We acknowledged that future studies should incorporate more detailed assessments of preventive practices, potentially through standardized behavioral scales or objective measures of adherence. Such enhancements would allow researchers to explicitly test risk compensation hypotheses and better understand the behavioral drivers of infection risk post-vaccination.

Differences in exposure risk

Vaccinated individuals might have engaged in more social or occupational activities, believing they were protected, thus increasing their exposure risk. This increased interaction could have contributed to higher infection rates despite vaccination. Studies have suggested that individuals who feel protected may partake in higher-risk behaviors [11].

The observed age difference, with younger participants showing higher infection rates, may reflect differences in exposure risk and behavior. Younger employees might be more engaged in roles requiring physical presence or interactions, leading to increased susceptibility. Additionally, they may participate in more social activities outside of work, thereby elevating their risk of contracting the virus. Understanding these demographic nuances is crucial for tailoring public health interventions within the SME sector.

Waning immunity

Waning immunity over time is another factor to consider. While mRNA vaccines were effective, their protective effect could diminish over time, especially against emerging variants [7,12]. Individuals with more vaccine doses may have been vaccinated earlier and could have experienced reduced immunity, making them more susceptible to infection if booster doses were not recent.

It is possible that immunological mechanisms, such as original antigenic sin or antibody-dependent enhancement (ADE), could contribute to the observed increase in infection rates among vaccinated individuals, although these phenomena remain largely theoretical in the context of COVID-19 vaccines. Kim et al. [13] highlighted the need for ongoing evaluation of long-term vaccine efficacy and potential immunological effects, including ADE, which may not be fully captured in early-phase clinical trials. Additionally, Arvin et al. [14] discussed ADE as a hypothetical concern in SARS-CoV-2, suggesting that certain antibodies might, under specific conditions, enhance viral entry rather than neutralize it. Current evidence does not confirm ADE in COVID-19 vaccines, but continued research is necessary to explore these and other potential immune responses in vaccinated populations.

Implications for future public health strategies

The findings from this study highlight important considerations for vaccination campaigns and public health interventions. Ensuring that vaccinated individuals continue to adhere to preventive measures during outbreaks is crucial. Public health messages should emphasize that vaccination is a critical tool, but does not provide absolute protection, especially over time or against new variants. Addressing behavioral risk compensation through effective communication strategies can enhance the overall effectiveness of vaccination programs [15].

Although we focused on employees of Japanese SMEs, our findings may be relevant to other occupational settings with similar workforce structures and exposure patterns. For example, industries with close physical proximity among workers, or those in countries with comparable public health measures, may find these results informative. However, differences in national health policies, cultural norms, and occupational regulations should be considered when interpreting the applicability of our findings in other regions or sectors.

Public big data and health center real-time information-sharing system (HERSYS)

The HERSYS on COVID-19 and similar public health databases can serve as valuable resources for future research, providing real-time data for analyses of infection trends and vaccine efficacy [16]. Leveraging such data may help validate findings and inform policy in the context of evolving epidemiological landscapes.

Need for longitudinal studies and validation across diverse populations

We recommended that future research employ longitudinal designs to better establish causal relationships between vaccination, behavioral changes, and infection outcomes over time. Additionally, expanding the scope of such studies to include diverse populations, industries, and cultural contexts would allow for a more comprehensive assessment of external validity. By investigating these factors across various demographic and epidemiological settings, future studies could validate and extend our findings, ultimately informing more effective public health and workplace strategies.

Limitations

This study has several limitations. The reliance on self-reported data may introduce recall bias and affect the accuracy of reported vaccination status and infection history. Because we relied on self-reported infection status and vaccination records, the potential for misclassification bias existed if some participants failed to report previous infections accurately, or if diagnostic criteria varied. Additionally, we acknowledged that the case-control design might have introduced survivor bias, as individuals who had severe outcomes may have been underrepresented if they were not available to participate. This could have skewed the results toward those who experienced milder infections or remained employed throughout the study. The study design also precluded precise survival time analysis, which could be performed in a cohort study, limiting our ability to assess temporal relationships and the duration of vaccine protection over time. Confounding variables, such as occupational exposure risk, socioeconomic status, and adherence to NPIs, were not fully controlled and may have influenced the results. Additionally, the study population is limited to employees of Japanese SMEs, which may not be representative of the general population, limiting the generalizability of the findings.

The simplicity of certain variables, such as behavioral factors, reflected the questionnaire’s inherent design, prioritizing efficient data capture over granular detail. Although this methodology supported a high response rate, it constrained the level of nuance obtained, underscoring the need for more granular instruments in future research. Also, as this study was designed as an analytical investigation rather than a confirmatory trial, no formal sample size calculation was undertaken. Furthermore, the study team adopted a complete-case approach, excluding participants lacking key data, which maintained internal consistency but may have introduced selection bias. Without imputation, readers should recognize that missing values could constrain generalizability and compromise validity. Future research should consider multiple imputation or similar methods for more rigorous handling of missing data.

A major limitation of this study is the inability to determine the temporal sequence between COVID-19 infection and vaccination due to the case-control study design. This uncertainty in causal ordering means we cannot definitively establish whether vaccination preceded infection, or vice versa. This could potentially explain the observed increase in ORs for COVID-19 infection with higher numbers of vaccine doses. For instance, individuals who became infected may have been more likely to receive additional vaccine doses afterward, thereby inflating the association between vaccine dose number and infection risk. While we compared unvaccinated individuals with those who received varying numbers of vaccine doses, it might have been more appropriate to compare each group with the preceding lower-dose group. However, even with this approach, the fundamental issue of uncertain causal direction remains unresolved. Recognizing this limitation highlights the need for longitudinal cohort studies that can establish clear temporal relationships between vaccination and infection, providing more definitive insights into the true causal effects.

## Conclusions

This study observed a higher incidence of COVID-19 infection among vaccinated individuals, escalating with the number of vaccine doses received. However, the inability to ascertain the temporal sequence between vaccination and infection necessitates a cautious interpretation of these findings. Behavioral factors, exposure risk, and potential waning immunity may have influenced post-vaccination infection rates. Elucidating these elements is imperative for optimizing public health strategies and vaccination initiatives. Future longitudinal studies with sophisticated designs are essential to establish definitive causal relationships and provide more conclusive insights.
